# Analysis of omega-3 fatty acid content of South African fish oil supplements

**DOI:** 10.5830/CVJA-2010-080

**Published:** 2011-11

**Authors:** Maretha Opperman, AJ Spinnler Benade, De Wet Marais

**Affiliations:** Functional Food Research Unit, Department of Agriculture and Food Science, Cape Peninsula University of Technology, Cape Town, South Africa; Functional Food Research Unit, Department of Agriculture and Food Science, Cape Peninsula University of Technology, Cape Town, South Africa; The Nutritional Intervention Research Unit, Medical Research Council of South Africa, Cape Town, South Africa

**Keywords:** supplements, eicosapentaenoic acid (EPA), docosahexaenoic acid (DHA), conjugated dienes (CD), fish oil

## Abstract

**Introduction:**

Substantial evidence describes the protective effects of marine-derived omega-3 (n-3) polyunsaturated fatty acids (PUFA) on cardiovascular diseases as well as many other conditions. Numerous fatty acid preparations are marketed for supplementing the Western diet, which is low in n-3 fats. Since these preparations may vary in their n-3 PUFA content, we tested 45 commercially available products on the South African market for their fatty acid composition.

**Method:**

Forty-five commercially available n-3 fatty acid supplements were analysed using gas–liquid chromatography to determine their fatty acid content.

**Results:**

More than half of the n-3 supplements available on the South African market contained ≤ 89% of the claimed content of EPA and/or DHA as stated on the product labels. To meet ISSFAL’s recommendation of 500 mg EPA + DHA/day can cost consumers between R2 and R5 per person per day (R60 to R150 p/p/month). Regarding rancidity, the majority of capsules contained conjugated diene (CD) levels higher than that of vegetable oil obtained from opened containers (three months) used for domestic cooking purposes, despite the addition of vitamin E as antioxidant.

**Conclusion:**

Since no formal regulatory structure for dietary supplements currently exists in South Africa, consumers depend on self-regulation within the nutraceutical industry for assurance of product quality, consistency, potency and purity. Our results indicate that more than half of the n-3 fatty acid supplements on the South African market do not contain the claimed EPA and/or DHA contents as stated on product labels, and they contained CD levels higher than that in unused vegetable oils obtained from opened containers used for domestic cooking purposes.

## Abstract

During the past few years, evidence-based nutritional research has confirmed the importance of omega-3 (n-3) fatty acids in reducing the risk for cardiovascular disease (CVD). Recently, there has been increasing understanding of the essentiality of n-3 fatty acids in reducing cardiovascular disease (CVD).^1^ N-3 fatty acids possess a wide range of biological effects, including reducing inflammatory responses, lowering triglyceride levels, reducing risk of arrhythmias, a small dose-dependent hypotensive effect, anti-atherogenic effects and a reduction in platelet aggregation, all of which contribute to protection against CVD.^2^

The n-3 fatty acids of particular concern for the prevention of CVD are eicosapentaenoic acid (EPA) and docosahexaenoic acid (DHA). EPA and DHA are very-long-chain n-3 fatty acids. The ability of the body to manufacture them from the precursors is limited and to ensure adequate supply of these fatty acids, their dietary source is of vital importance.^3^ These fatty acids are predominantly found in fish and fish oils.^4^ Fatty fish such as herring, mackerel, tuna, salmon and trout are rich dietary sources of EPA and DHA.^1^

Since solid and voluminous scientific backing for the health benefits of n-3 fatty acids exists, a high level of public awareness and acceptance of n-3 fatty acids is becoming more apparent. Considering the fact that it is not always possible to consume adequate amounts of n-3 fatty acids through the diet, the interest in n-3 fatty acid supplements has soared. The position of the American Dietetic Association^1^ on nutritional supplements is that supplements can help some individuals meet their nutrient needs when their diet is inadequate due to different circumstances, including being in an ‘at-risk’ life-stage group for a nutrient deficiency. Examples of such risk groups include the geriatric population, pregnant women, individuals with compromised nutritional status and those with a limited variety in food selection, which prevents achieving nutrient adequacy.

However, although research has shown beneficial effects with increased intakes in n-3 fatty acids, excess intakes can be just as harmful as a deficiency and therefore n-3 fatty acids should be supplemented with caution. Detrimental effects of excess intake of n-3 fatty acids in healthy populations include depression of the immune function, bleeding and increased risk of haemorrhagic stroke, as well as increased lipid peroxidation resulting in oxidative damage to various tissues.^5^ At this point in time there is not sufficient supporting data to establish an upper limit (UL) or safe intake of n-3 fatty acids, however, the FDA has ruled that intakes of up to 3 g/d of marine n-3 fatty acids are generally recognised as safe (GRAS) for inclusion in the diet.^6^

Undesirable effects from consuming n-3 fatty acids have also been identified in a few selected populations. It has been suggested that diabetics or individuals with impaired glucose tolerance must use n-3 fatty acid supplements with caution since it might have detrimental effects on glucose homeostasis. Increased incidences of nosebleeds have also been reported in individuals with hypercholesterolaemia with n-3 fatty acid supplementation. Furthermore, simultaneous intake of n-3 fatty acids with medication such as aspirin and warfarin will excessively prolong bleeding times in individuals using anti-coagulants.^5^

Recommendations regarding the daily intake of n-3 fatty acids vary between 400 and 1 000 mg EPA + DHA in the form of food or supplements.^7^-^13^ Nevertheless, it is still not known what the optimal daily intake of EPA and/or DHA should be and how these fatty acids affect the metabolism of 18-carbon fatty acids and longer n-6 and n-3 fatty acids, and whether this might result in any adverse health outcomes. Based on the available evidence, n-3 fatty acid supplements provided in the appropriate dose would be expected to confer health benefits, especially in individuals who do not eat fish.^1^

To obtain these suggested daily intakes might be challenging for the consumer if only fatty fish is eaten, since ample amounts of about 220 to 240 g fatty fish (e.g. salmon) must be consumed weekly to reach a daily intake of 500 mg EPA + DHA.^14^ Therefore, fish oil preparations may be helpful to reach the daily recommended n-3 fatty acid intake. However, the actual long-chain n-3 fatty acid content of fish may fluctuate greatly, even within a single species, and is dependent on geographic origin, season and preparation.^15^,^16^

In a recent study^17^ in Belgium, 15 food supplements formulated as soft capsules containing n-3 fatty acids were evaluated by desegregation, determination of peroxide values and assay of the n-3 fatty acid content. All the products contained purified fish oil rich in n-3 fatty acids, mainly EPA and DHA, and were available as triglycerides, ethyl esters or free fatty acids. Seven of the 15 food supplements deviated from one or more of the criteria with regard to the recommended peroxide value and the content of one or more of the fatty acids.

In an analysis of n-3 fatty acids in fish oil supplements conducted in Austria, nine supplements were analysed using capillary gas chromatography to determine the n-3 fatty acid content. In comparison with the manufacturer’s information, four supplements did not differ significantly from the concentrations given, whereas four supplements contained substantially more EPA and DHA than stated on the label. One of the manufacturers did not declare the amount of n-3 fatty acids in the product. Regarding manufacturer’s recommendations for daily intake, one of the supplements exceeded the amount recommended by healthcare organisations. Five of the nine supplements lacked more than 30% EPA and DHA to meet the American Heart Association’s recommendations. Additionally, two supplements failed to achieve the range of 0.5 to 1.8 g n-3 fish oil found to be effective in most studies.^18^

Currently, no similar existing data are available for n-3 fatty acid supplements offered on the South African market. Although n-3 fatty acids in the form of supplements appear to be the safer and more controllable way of consuming n-3 fatty acids, all preparations rely on fish oil as a source of EPA and DHA, which may show the same variability as the natural product these preparations are derived from. Additionally, South African consumers have grown accustomed to the high quality inherent in the manufacture of conventional medicinal products and usually accept without question the consistency, purity and potency of prescription and non-prescription medications. Consequently, consumers have little reason to doubt the package label claims on conventional medications.

However, a similar claim cannot be made for dietary supplements in South Africa since no regulatory structure for dietary supplements currently exists. As a result, consumers of nutritional supplements must depend on self-regulation within the nutraceutical industry for assurance of product quality, consistency, potency and purity. Reliable and trustworthy nutritional information is vitally important for the consumer since misleading nutritional information can lead to errors in daily dosage, with serious dose-related side effects.

The aim of this study was therefore to analyse the fatty acid content, composition and the level of rancidity of commercially available n-3 supplements on the South African market. The objectives of the analyses were to compare analysed EPA and DHA content of capsules to manufacturers’ labelling information, to compare the number of capsules and price of supplements to meet international recommended dietary intakes, to supply an indication of the EPA to DHA ratio of commercially available capsules, and to measure the conjugated diene content (early indication of lipid peroxidation) in n-3 fatty acid supplements on the South African market. In addition, the mercury content of all the products was determined.

## Methods

The fatty acid content, composition and conjugated diene content of 45 commercially available n-3 fish oil supplements were analysed. Brands analysed, in no particular order, included: Amipro®, Biogen®, Equazen®, Vital®, Clicks®, Clicks Health Basics®, Bettaway®, Thinkwell®, AddVance®, NutriLida®, Dischem®, Golden Products®, Health Balance®, Holstix Fish®, AddAway®, Tslim Plus®, Amway Nutrilite®, Rejuvenesse®, The Real Thing®, Solal®, Durbell®, Natrodale®, Optimega®, Revite®, Vitaforce®, Mum Omega®, Preg Omega®, OmegaCare®, Brain Child®, Bioter Health® and Unique Formulations®.

The conjugated diene (CD) content of oils represents an early stage of oxidation (rancidity). The content of conjugated dienes in the 45 n-3 capsules were determined spectrophotometrically as described by Recknagel and Glende.^19^ Since no CD reference values were available for oils, the CD content of fresh, unopened sunflower, olive palm and canola oil were used against which to compare the supplements’ CD contents. The CD contents of sunflower, olive and canola oil were 4.28, 6.68 and 8.28 μM, respectively. The CD contents of sunflower, olive and canola oil of which half of the contents had already been used were 16.8, 18.2 and 18.7 μM, respectively.

The content of mercury in the 45 n-3 capsules was determined by atomic absorption spectroscopy according to the method of Aduna de Paz *et al*.^20^ Samples were digested with nitric acid (HNO_3_) and hydrogen peroxide (H_2_O_2_) in a microwave oven (Milestone MLS-1200 MEGA, Milestone GmbH) equipped with a high-pressure rotor for six teflon vessels. Total mercury (Hg) was determined by cold vapour generation on a Thermo atomic absorption spectrophotometer (SOLAAR M Series) coupled to a vapour generator (Thermo model VP100, Thermo Scientific), using SnCl_2_ as the reductant. Standard quality-control procedures were adopted throughout. Spiked samples gave an average recovery of 94.1%.

A Varian model 3300 chromatograph was used and fitted with a BPX-70 fused silica capillary column (30 m × 0.32 mm i.d., 0.25-mm film thickness). The injector and flame ionisation detector was at 240 and 280°C, respectively. The column temperature was programmed from 160 to 220°C at a rate of 3°C per minute. A hydrogen column flow rate of 30 cm.sec^-1^ was used.

The method of fatty acid extraction and methylation as described by Christie^21^ was slightly modified to suit our analytical requirements: 100 mg of sample was dissolved in 20 ml chloroform/methanol (2:1); 200 μl of this solution was then dried under a stream of nitrogen and 2 ml of a fresh solution of 5% sulphuric acid in methanol was added in glass-stoppered tubes and incubated in a water bath at 70°C for two hours. After cooling, 1 ml of distilled water and 2 ml of hexane were added to the sample and vortexed for one minute. The hexane phase was allowed to separate and was then transferred to a glass tube to be evaporated under a stream of nitrogen at 40°C.

The residue was then re-dissolved in 50 μl carbon disulphide (CS_2_) and 1 μl was injected into the gas chromatograph. Heptadecanoic acid was used as an internal standard. Butylated hydroxy toluene was added as an antioxidant to all samples. The EPA and DHA content of the samples were quantified and expressed as a percentage of EPA and DHA of total fatty acid content.

One of the objectives of this research was to compare the EPA and DHA contents, as claimed by manufacturers on the supplement label, with the actual measured contents. An acceptable range between 90 and 110% of the manufacturers’ claimed content for EPA and DHA was proposed. Therefore, supplements containing ≤ 89% of the claimed EPA and/or DHA content were perceived as substandard supplements, while those containing more than 110% were considered to be excessive. The coefficient of variance (CV) for the analysis of EPA and DHA was 2%.

## Results

**Fig. 1.**
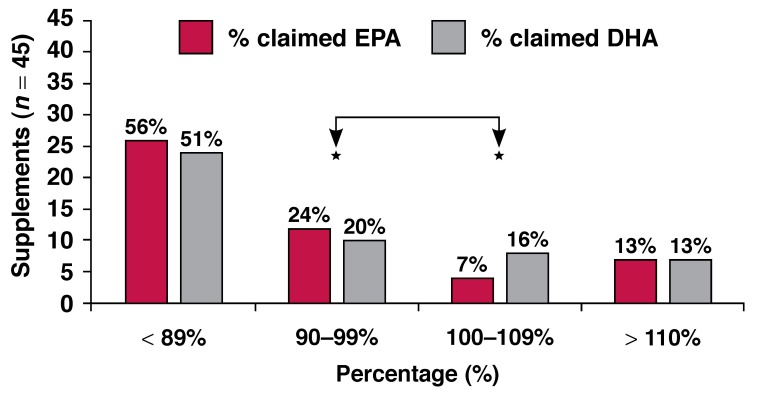
Percentage of claimed EPA and/or DHA content. *Acceptable range = 90–110%.

Comparison with the manufacturers’ labelling information (Fig 1) showed that 56% (*n* = 25) and 51% (*n* = 23) of the supplements failed to meet the lowest range of 89% for EPA and DHA concentrations, respectively. Only 31% (*n* = 14) of the EPA and 36% (*n* = 16) of the DHA contents of supplements were within the acceptable range of 90 to 110%. Thirteen per cent (*n* = 6) of the preparations held more EPA than stated, while a similar number (13%; *n* = 6) of supplements had a higher DHA content than indicated.

## Number of capsules and price to reach recommendations

**Fig. 2.**
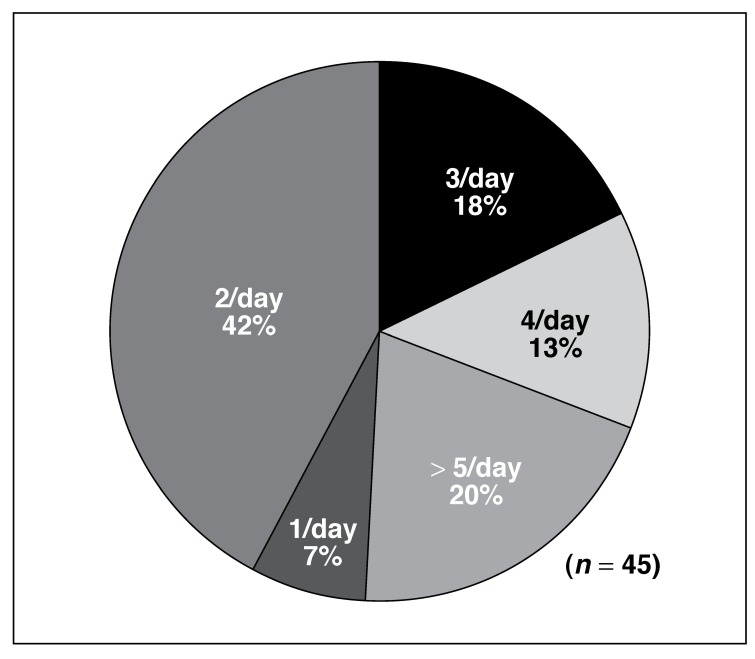
Summary of the number of capsules needed to meet ISSFAL^14^ (500 mg EPA + DHA per day) recommendation.

**Fig. 3.**
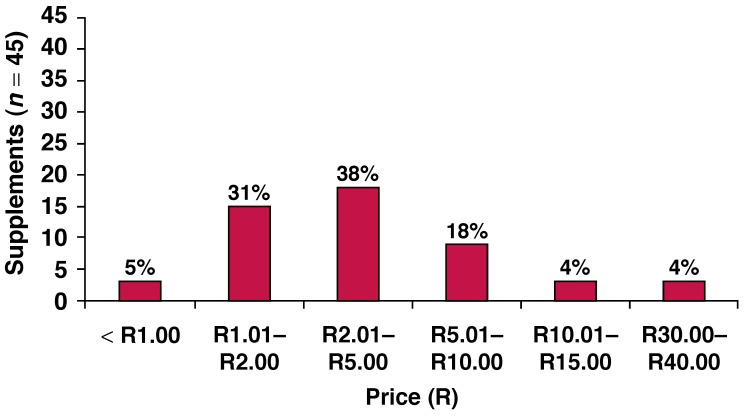
Price (R) to achieve daily intake of 500 mg EPA + DHA.

Currently there are no South African daily dietary intake recommendations for n-3 fatty acids. However, manufacturers of n-3 fatty acid supplements suggest a daily dosage of capsules on their labels, with no indication of the basis on which these recommendations were made. For the purpose of this publication, the International Society for the Study of Fatty Acids and Lipids (ISSFAL)^14^ recommendation of 500 mg EPA + DHA per day for the prevention of cardiovascular disease was applied as guideline. Fig. 2 provides a summary of the number of capsules needed to reach ISSFAL’s^12^ recommendations while Fig. 3 highlights the cost (ZAR) to achieve a daily intake of 500 mg EPA + DHA/day.

Forty-two per cent (*n* = 19) of supplements were able to supply 500 mg EPA + DHA/day with the administration of two capsules per day, while only 7% (*n* = 3) of supplements could provide the recommended intake by consumption of one capsule per day. In 20% (*n* = 9) of the supplements, more than five capsules per day had to be ingested daily to meet the ISSFAL^14^ recommendation.

The majority (38%; *n* = 17) of the supplements varied between R2.01 and R5.00 per day to meet the ISSFAL^14^ recommendation of 500 mg EPA + DHA/day. This represents an amount of R60.30 to R150.00 per month. Less than a third (31%; *n* = 14) of the supplements were priced between R1.01 and R2.00 per day (R30.30 to R60.00 per month). Some supplements (4%; *n* = 1) even cost up to R30.00 to R40.00 per day to supply a 500-mg EPA + DHA dosage.

## EPA to DHA ratio

Fish oils from different sources contain variable mixtures of EPA and DHA. Most commercially available fish oils contain a proportion of 2:1 EPA to DHA.^22^ Regarding the EPA to DHA ratio in South African n-3 fatty acid supplements, most of the studied supplements (40%; *n* = 18) had an EPA:DHA ratio of 1.51–2.0:1, while 36% (*n* = 16) of supplements had a 2.1–2.5:1 EPA:DHA ratio. Only a few (13%; *n* = 6) supplements had a higher DHA:EPA ratio (EPA:DHA ratio < 0.5) (see Table 1).

**Table 1.** EPA To DHA Ratio In South African N-3 Fatty Acid Supplements

*Ranges*	*EPA:DHA ratio (n)*	*EPA:DHA ratio (%)*
< 0.5:1	6	13
– 1.5:1	2	4
1.51–2.0:1	18	40
2.1–2.5:1	16	36
2.51–3.0:1	0	0
3.0–3.5:1	1	2
> 5:1	2	4

## Conjugated dienes

**Fig. 4.**
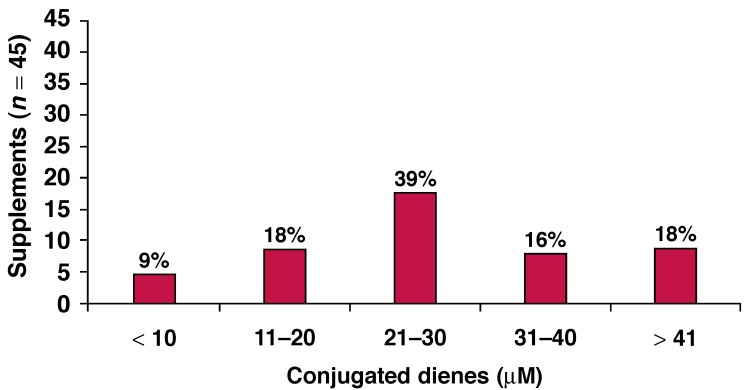
Conjugated diene content of South African n-3 fatty acid supplements.

The majority (73%; *n* = 33) of commercially available n-3 fatty acid supplements had a CD content higher than 21 μM. Only 27% (*n* = 12) of the n-3 fatty acid preparations contained a CD content of less than 20 μM, while barely any supplements (*n* = 4; 9%) contained a CD content comparable to fresh, unopened oils (see Fig. 4). These values were measured notwithstanding the presence of added vitamin E as an antioxidant.

## Mercury contamination

Mercury was virtually absent from the oils in the supplements and was therefore not of any health concern in these samples.

## Discussion

An extensive variety of n-3 fatty acid supplements are available to the South African consumer, however, our results have shown that supplements vary to a large extent in terms of claimed and measured EPA and/or DHA content, levels of fatty acid oxidation, EPA to DHA ratio, as well as numbers of capsules and price to meet international dietary recommendations. When comparing claimed to measured contents of EPA and DHA in South African n-3 fatty acid supplements, it is of concern that information appearing on almost two-thirds of these supplements’ labels was not a true reflection of the actual contents of the supplements. It was decided to compare supplements against an arbitrary 90 to 110% standard. In other words, supplements’ label information was considered as a truthful reflection of the measured content if the EPA and/or DHA content analyses were between 10% less or 10% more, compared to the claimed content.

More than half of commercially available South African n-3 fatty acid supplements failed to attain 90% of the claimed contents of EPA or DHA or both, while approximately 15% of supplements contained more than 110% of the claimed contents of EPA or DHA or both. Since the typical Western diet is characterised by a low n-3 and very high n-6 fatty acid intake, many consumers rely on supplements to increase their daily n-3 intakes. Unfortunately, if unreliable information is published on labels, consumers are supplied with misleading information, leading to erroneous dosages, with subsequent consequences.

If a supplement contains less n-3 fatty acids than claimed, consumers waste their money without optimal improvement of their n-3 fatty acid status. In contrast, excess n-3 fatty acid intakes can be just as detrimental as a deficiency. Adverse effects of excess intake of n-3 fatty acids in healthy populations include suppression of the immune function, bleeding and increased risk of haemorrhagic stroke, as well as increased lipid peroxidation, resulting in oxidative damage to various tissues. Furthermore, simultaneous intake of n-3 fatty acids with medication such as aspirin and warfarin will excessively prolong bleeding times in individuals using anti-coagulants.^5^ The FDA has ruled that intakes of up to 3 g/d of marine n-3 fatty acids are generally recognised as safe (GRAS) for inclusion in the diet.^5^

Regarding the number of capsules needed to meet optimal n-3 fatty acid intakes, our results indicate that only a few supplements were able to provide the daily need in one capsule. Some supplements even required a dosage of more than five capsules to meet international recommendations. In addition to this, our analyses have shown that the majority of n-3 supplements on the South African market were priced between R2.01 and R5.00 per day to meet the ISSFAL recommendation of 500 mg EPA + DHA per day. This represents an amount of R60.30 to R150.00 per individual per month. To provide a family of four with the daily recommended intake of 500 mg EPA + DHA adds up to between R242.40 and R600.00 per family per month.

Since malnutrition, especially in poverty-stricken areas, is a major health problem in South Africa, it can be accepted that many people have either a marginal or deficient n-3 fatty acid status. Considering the current financial situation in South Africa, in combination with a large part of the South African population living in poverty, this amount is substantial in terms of monthly expenses for the average South African family. Hence, it is impossible for the average South African to consume an n-3 fatty acid supplement on a regular basis. Some supplements are even more expensive and can cost up to R1 060 per person per month to meet the recommended intake of 500 mg EPA + DHA per day.

The ratio of EPA to DHA in n-3 supplements has become an important point of discussion. Gorjão *et al*.^22^ reported that most commercially available fish oils present with a 2:1 ratio of EPA to DHA, while numerous cold-water oily fish sources have higher DHA to EPA ratios.^23^ According to Kris-Etherton *et al*.,^16^ the majority of commercially available n-3 fatty acid supplements in the United States provide 180 mg EPA and 120 mg DHA per capsule, representing a ratio of 1.5:1 EPA to DHA. EPA and DHA have different effects on various health aspects and under certain conditions it seems that a higher DHA to EPA ratio is preferable.

In the brain, DHA is the main n-3 polyunsaturated fatty acid,^24^ and the importance of DHA in neural and visual development and function, especially during pregnancy, lactation and infancy,^25^,^26^ is well documented. Additionally, deficits in DHA appear to contribute to inflammatory signalling, apoptosis and neuronal dysfunction in the progression of Alzheimer’s disease (AD), a common and progressive age-related neurological disorder unique to structures and processes of the human brain.^27^

With regard to cell function, Gorjão *et al*.^22^ reported that some studies have shown that EPA and DHA have diverse effects on cell functions such as leukocyte functions. EPA and DHA also modulate the expression of genes in lymphocytes differently, and affect the activation of intracellular signalling pathways involved with lymphocyte proliferation in a different way, therefore necessitating different EPA to DHA ratios to ensure optimal function.

Mori and Woodman^28^ compiled a review on the independent effects of EPA and DHA on risk factors for cardiovascular disease in humans. From their report, it seems that EPA and DHA have diverse haemodynamic and anti-atherogenic effects. According to Mori and Woodman,^28^ both EPA and DHA are effective in reducing serum triglyceride levels but only DHA has the ability to increase high-density lipoprotein cholesterol (HDLC). DHA also increases low-density lipoprotein (LDL) particle size, a potential anti-atherogenic effect. Neither EPA nor DHA show any effects on total cholesterol, while it appears that DHA is more effective in reducing blood pressure and heart rate when compared to EPA.

However, most clinical data available on the cardiovascular effects of n-3 fatty acids used a combination of EPA + DHA supplementation. Future research studies should therefore assess the individual effects of EPA and DHA in a variety of clinical settings and target populations, before decisions can be made on specific ratios of EPA to DHA in supplements and food fortified with either EPA or DHA.

Conjugated dienes (CDs) contain two or more double bonds and are formed during the oxidation process of unsaturated fatty acids to ensure a more stable radical.^30^ CDs are used to determine primary oxidation products and therefore provide an early indication of the levels of lipid oxidation.^31^ Although primary oxidation products such as CDs have no colour or flavour of their own, they can readily be decomposed to secondary products such as aldehydes, ketones and alcohols. These secondary oxidation products have distinctive flavours and contribute to the offensive taste of decomposed seafood and marine oils.^31^

Considering the CD content of commercially available South African n-3 fatty acid supplements, it seems that the majority contain high amounts of primary oxidation products. No clear relationship could be established between the expiry dates and the CD content of the n-3 supplements. These results therefore suggest that a considerable variation exists in the quality of the fish oil present in n-3 capsules in South Africa. This indicates that the oils present in many of the supplements are in the first stages of rancidity and hence negatively influence the quality of the product that consumers buy.

An additional health concern related to fish and fish oil supplements is that some species of fish may contain considerable levels of heavy metals such as methyl mercury.^8^ Methyl mercury may be present at low levels in fresh waters and oceans but tends to concentrate in the aquatic food chain such that levels are generally highest in older, larger, predatory fish and marine mammals. Fish and seafood are a major source of human exposure to methyl mercury.

Methyl mercury has a relatively long half-life in human tissue and can accumulate in individuals who consume contaminated fish and fish oils on a regular basis. Skinning and trimming is usually recommended to reduce exposure to contaminants but because methyl mercury is distributed throughout the muscle, skinning and trimming does not significantly reduce mercury concentrations in fillets. Pregnant and lactating women as well as children are generally advised against the consumption of shark, swordfish, king mackerel and tilefish, since these species may contain higher levels of mercury.^32^ Our analysis has shown that mercury was virtually absent in the oils present in the South African samples and it is therefore not of any health concern.

It is undoubtedly the responsibility of manufacturers to provide accurate information on supplement labels to protect the consumer against misleading health and nutrient claims, to ensure the safety of the consumer and to guarantee a high-quality, consistent product. However, in South Africa this does not seem to be the case.

Possible reasons for substandard supplements on the South African market may include: poor quality of imported fish oil, seasonal differences in EPA and/or DHA concentrations of imported fish oils, lack of proper labelling legislation of food supplements, inappropriate handling of fish oil when harvested, improper storage conditions of both fish oil and supplements, oxidation of fatty acids, ineffective quality assurance by supplement manufacturers, and infrequent or poor batch-control analyses. If these issues are not addressed and legislation on food supplements is not enforced, South African consumers will have to deal with substandard dietary supplements.

## Conclusion

More than half of the n-3 supplements available on the South African market contained less than the amount of EPA and/or DHA content as claimed on the labels of the products, which has considerable cost implications for the consumer. Early indicators of rancidity in the majority of capsules suggest a wide variation in the quality of the marine oils present in the n-3 capsules available on the South African market. This is despite the addition of vitamin E as antioxidant. South African n-3 fatty acid supplements appear to be virtually free of methyl mercury.
